# Lemongrass (*Cymbopogon citratus*) oil: A promising miticidal and ovicidal agent against *Sarcoptes scabiei*

**DOI:** 10.1371/journal.pntd.0008225

**Published:** 2020-04-06

**Authors:** Meilin Li, Buming Liu, Charlotte Bernigaud, Katja Fischer, Jacques Guillot, Fang Fang

**Affiliations:** 1 Parasitology Department, College of Animal Science and Technology, Guangxi University, Nanning, China; 2 Guangxi Key Laboratory of Traditional Chinese Medicine Quality Standards, Guangxi Institute of Traditional Medical and Pharmaceutical sciences, Nanning, China; 3 EA 7380 Dynamyc, Faculté de Médecine de Créteil, UPEC, EnvA, USC Anses, Ecole nationale vétérinaire d’Alfort, Maisons-Alfort, France; 4 Dermatology Department, AP-HP, Hôpital Henri Mondor, Université Paris-Est, Créteil, France; 5 Cellular and Molecular Biology Department, Infectious Diseases Program, QIMR Berghofer Medical Research Institute, Brisbane, Australia; Hitit University, Faculty of Medicine, TURKEY

## Abstract

**Background:**

Essential oils may represent an alternative strategy for controlling scabies, a neglected tropical disease caused by the infestation of mite from the species *Sarcoptes scabiei*. Lemongrass (*Cymbopogen citratus*) oil is reported to possess pharmacological properties including antiparasitc, antioxidant, antimicrobial and anti-inflammatory. The aim of the present study was to assess the potential efficacy of lemongrass oil against the mites and eggs of *Sarcoptes scabiei*.

**Methodology/Principal findings:**

Mass spectrometry analysis confirmed that the main component presented in lemongrass oil was citral. Lemongrass oil at concentrations of 10% and 5% killed all *Sarcoptes* mites within 10 and 25 min, respectively. The median lethal concentration value was 1.37%, 1.08%, 0.91%, 0.64%, and 0.48% at 1, 3, 6, 12, and 24 h, respectively. Lemongrass oil at all concentrations (10%, 5%, 1%, 0.5%, 0.1%) was able to significantly decrease the hatching rate of *Sarcoptes* eggs.

**Conclusions/Significance:**

Lemongrass oil should be considered as a promising miticidal and ovicidal agent for scabies control.

## Author summary

Scabies is a parasitic infestation which affects about 455 million people annually, with a particularly high prevalence in low income tropical regions. The disease is frequently complicated by bacterial infections. Currently available treatments do not meet the ideal profile which includes miticides, ovicidal, antibacterial and anti-inflammatory/antipruritic properties. *Cymbopogon citratus* is a plant widely cultivated in tropical and subtropical regions. The essential oil from *C*. *citratus* is known as lemongrass oil with reported antimicrobial and anti-inflammatory properties. We conducted experiments to assess the miticidal and ovicidal efficacy of lemongrass oil against *Sarcoptes* mites collected from naturally infected rabbits in China. The results demonstrated that lemongrass oil can kill the motile stages of *Sarcoptes* mites effectively and presented a significant ovicidal activity. Considering all these properties, lemongrass oil should be considered a promissing miticide against scabies.

## Introduction

Scabies is a contagious skin disease caused by the infestation of *Sarcoptes scabiei* mites. The clinical symptoms include intense itch, erythema, and more severe complications due to secondary bacterial infections [1]. Scabies was added to the WHO list of neglected tropical diseases in 2017, which highlights the substantial global health and economic burden, especially in low income populations [2,3].

Control of scabies is predominantly performed by the use of synthetic miticides with topical agents (e.g. permethrin, benzyl benzoate, malathion and crotamiton) and oral ivermectin. Unfortunately, most chemical miticides are suboptimal, as they are either associated with moderate to severe side effects and/or require multiple treatments due to limited ovicidal activity [4,5]. In addition, resistance to the mostly used miticides has emerged [6]. Novel drug with new modes of action could slow down the emergence of resistance if used alternated with existing drugs [7]. Studies have shown that essential oils such as clove, tea tree and palmorosa are effective against the motile stages of *Sarcoptes* mites [8–10]. As a topical therapy, 5% tea tree oil has been proposed for scabies treatment in Australia [11]. However, none of these studies have tested the ovicidal activity of these oils.

*Cymbopogon citratus* (DC.) Stapf 1906, is a plant widely cultivated in tropical and subtropical regions. The essential oil from *C. citratus* is known as lemongrass oil, with citral as the major component [12]. Lemongrass oil is reported to possess a variety of pharmacological properties including antiparasitic [13], antioxidant [14], antimicrobial [12], and anti-inflammatory [15]. Lemongrass oil was also reported to show miticididal effect against a plant pathogen (*Tetranycchus urticae*) [16] and house dust mites [17]. However, its activity against *S. scabiei* has not been described so far. In this study, we extracted and analyzed the essential oil of *Cymbopogon citratus* collected in Southern China and further assessed its miticidal and ovicidal efficacy against *S. scabiei* from rabbits.

## Methods

### Ethics statement

Before the beginning of the study, we contacted farm owners and obtained their permission to have the infected rabbits involved. The study protocol was approved by the ethics committee of Guangxi University (approval no: GXU2019-019). The animals were handled in accordance with guidelines established by the French and European regulations for care and use of animals for scientific purposes.

### Plant material and extraction

*Cymbopogon citratus* plants were collected from Tianlin County of Guangxi province, China. The lemongrass oil was obtained by hydrodistillation of fresh leaves from the plant, and was dried with anhydrous sodium sulfate. For hydrodistillation, 250g of fresh lemongrass leaves were cut into small pieces and placed in a 1L flask containing 400mL of distilled water. They were hydrodistilled for 5 h using a Clevenger-type apparatus according to the method described by Guenther (1950)[18]. The yield of essential oil was calculated using the following formula: Essential oils yield (%) = net weight of oils (g) / total weight of fresh leaves (g) × 100. The extraction yield was 0.4%. The oil was stored in dark glass bottles and kept at 4°C until use.

### Essential oil analysis

The components of lemongrass oil were analyzed by gas chromatography/mass spectrometry (GC/MS) using an Agilent 5977A Series GC/MSD System. Analytes were separated with a HP-5MS quartz capillary column (30 m x 0.25mm x 0.25μm). The analysis was performed using the following temperature program: initial temperature at 60°C, held for 2 min; and increased at the rate of 5°C /min to 90°C, held for 2 min; and increased at the rate of 5°C /min to 130°C; then increased at the rate of 10°C /min to 150°C, held for 1 min; and increased at the rate of 10°C /min to 200°C, held for 2 min; finally increased at the rate of 10°C /min to 240°C. Helium was used as the carrier gas (1 mL/min) at a split ratio of 200:1. The ion source temperature was 250°C, and mass spectra were obtained in EI-scan mode at 70 eV electron energy. The sector mass analyzer was set to scan from 35 to 500 amu. Identification of compounds was based on a comparison of mass spectra of each peak with those of authentic samples in a mass spectrum library. The percentages of compounds were calculated by the area normalization method.

### Collection of mites and eggs

*Sarcoptes scabiei* mites and eggs were collected from the crusts of naturally infested New Zealand white rabbits in a rabbit farm in Nanning, Guangxi Province, China. The crusts were placed in Petri dishes and transported to the laboratory within 1 hour. Living mites of all stages (adults, nymphs and larvae) and eggs were isolated one by one with a needle for test under a stereomicroscope.

### *In vitro* evaluation of miticidal activity

The lemongrass oil was diluted to five concentrations (10%, 5%, 1%, 0.5%, and 0.1%) with paraffin oil. A contact bioassay was performed according to a previously described method [10]. Twenty mites of all motile stages (adults, nymphs and larvae) were placed into a plastic sterile Petri dish (3 cm in diameter). Then, 0.5 mL of each solution was added to the Petri dishes. Negative control dishes were treated with 0.5mL paraffin oil and positive control dishes were treated with 0.5mL 25% benzyl benzoate (Aladdin, China). All Petri dishes were maintained at room temperature (25°C) and 70% relative humidity. For each concentration of lemongrass oil, as well as negative and positive controls, a total of five replicates were performed. Mite mortality was monitored and evaluated under a stereomicroscope every ten minutes for 1 h, and then after 3 h, 6 h, 12 h, and 24 h of exposure to treatment. Mites showing persistent immobility, even after repeated agitation with a needle, were considered dead.

### *In vitro* evaluation of ovicidal activity

Lemongrass oil was used in five concentrations (10%, 5%, 1%, 0.5%, and 0.1%) to test the activity against *Sarcoptes* eggs. A filter paper was placed at the bottom of a Petri dish and impregnated with each solution (0.3 mL). Negative control filter papers were treated with 0.3 mL paraffin oil, and positive control filter papers were treated with 0.3mL of 25% benzyl benzoate (Aladdin, China). After they were dried in a fume hood for 10 min, 20 eggs at early stages of development were placed on the filter paper. All Petri dishes were placed in a humidity chamber (≥70% relative humidity) at 35°C. For each concentration of lemongrass oil, as well as negative and positive controls, a total of three replicates were performed. The hatchability of eggs was determined under a stereomicroscope 5 days after the treatments [5]. Eggs were considered dead if they failed to hatch after 5 days.

### Data analyses

Data of above experiments were analyzed by SPSS 20.0 software and were expressed as the mean ± SD. The data were analyzed with one-way analysis of variance (ANOVA) followed by LSD (Least Significant Difference). Probit regression analysis was used to calculate the median lethal concentration value (LC_50_) and median lethal time value (LT_50_).

## Results

### Essential oil analysis

The chemical composition of lemongrass oil is presented in Table 1. GC-MS analysis showed that the main compounds were geranial (trans-citral, citral A) (37.40%), neral (cis-citral, citral B) (31.97%) and myrcene (15.65%).

**Table 1:** Chemical composition of lemongrass (*Cymbopogon citratus*) oil collected in Guangxi, China. The components were analyzed by gas chromatography/mass spectrometry (GC/MS) with an Agilent 5977A Series GC/MSD System.

Compounds	Retention time (min)	Percentage (%)
6-Methyl-5-Hepten-2-One	5.9	0.76
Myrcene	6.0	15.65
Limonene	6.9	0.65
Octatriene	7.1	0.61
β-Octatriene	7.4	0.36
Piperitenone	8.7	0.12
Linalool	8.9	1.12
β-Citronellene	10.6	0.14
Citronellal	10.8	0.16
Cis-Carveol	11.7	0.15
Trans-Carveol	11.8	1. 55
Citronellol	12.3	1.10
Geraniol	13.4	1.55
Neral	13.9	31.97
Nerol	14.3	2.66
Geranial	14.9	37.40
2-Undecanone	15.5	0.67
Caryophyllene	19.0	0.30
α-Bergamotene	19.3	0.17
2-Tridecanone	20.6	0.89
Cadinene	21.0	0.10
Aromadendrene	21.2	0.13
Isoaromadendrene	22.8	0.11
Selina-6-en-4-ol	23.0	0.54
Cadinol	23.7	0.10
2-Pentadecanone	24.2	0.17

### *In vitro* evaluation of miticidal activity

Compared to paraffin oil (negative control), lemongrass oil showed a significant effect against *Sarcoptes* mites, in a dose- and time-dependent manner. At the concentration of 10% and 5%, lemongrass oil killed all the mites within 10 and 25 min, respectively. The miticidal efficacy was significantly better than the positive control of 25% benzyl benzoate which with a mean mortality rate of 66 min. The mean mortalities at 24 h of 1%, 0.5% and 0.1% were 100%, 48.3% and 12%, respectively (Table 2). All concentrations of lemongrass oil showed a good miticidal efficacy compared to the negative controls (P<0.01). Further analyses indicated that the LC_50_ value of lemongrass oil at 1 h, 3 h, 6 h, 12 h and 24 h was 1.37%, 1.08%, 0.91%, 0.64% and 0.48%, respectively (Table 3). The LT_50_ value for lemongrass oil at the concentration of 10%, 5%, 1%, 0.5% and 0.1% was 4 min, 9 min, 4 h, 25.5 h and 42.5 h, respectively (Table 4)

**Table 2:** Miticidal activity of lemongrass (*Cymbopogon citratus*) oil against *Sarcoptes scabiei in vitro*.

Oil concentration	Mean mortality (%)± SD				
	1h	3h	6h	12h	24h
10%	100^a^	100^a^	100^a^	100^a^	100^a^
5%	100^a^	100^a^	100^a^	100^a^	100^a^
1%	11.9±7.9^c^	37.5±20.7^b^	63.9±13.5^b^	100^a^	100^a^
0.5%	0^d^	3.4±3.9^c^	3.4±3.9^c^	18.3±6.4^b^	48.3±11.4^b^
0.1%	0^d^	0^d^	0^d^	0^d^	12.0±8.4^c^
paraffin oil	0^d^	0^d^	0^d^	0^d^	0^d^
25% benzyl benzoate	78±8.4^b^	100^a^	100^a^	100^a^	100^a^

^a,b,c,d^Values followed by different letters within the same column are statistically significant (P<0.01).

**Table 3:** Probit regression analysis of the toxicity (LC_50_) of lemongrass (*Cymbopogon citratus*) oil against *Sarcoptes scabiei in vitro*.

Time	Regression line	LC_50_ (%) (95%FL*)	Pearson Chi-square
1h	Y = 3.107X-4.267	1.373 (1.144–73.927)	2.568
3h	Y = 2.908X-3.151	1.084 (0.970–1.295)	9.015
6h	Y = 3.736X-3.390	0.907 (0.830–1.002)	5.308
12h	Y = 6.845X-4.352	0.636 (0.581–0.727)	1.607
24h	Y = 3.347X-1.620	0.484 (0.410–0.564)	8.022

**Table 4:** Probit regression analysis of the toxicity (LT_50_) of lemongrass (*Cymbopogon citratus*) oil against *Sarcoptes scabiei in vitro*.

Oil concentration	Regression line	LT50 (95%FL*)	Pearson Chi-square
10%	Y = 33.879x-2.107	4.040 min#	7.230
5%	Y = 18.124x-2.614	8.926 min#	4.980
1%	Y = 0.293x-1.186	4.047 h (3.528–4.548)	32.604
0.5%	Y = 0.061x-1.566	25.512 h (23.015–28.480)	13.350
0.1%	Y = 0.052x-2.224	42.478 h#	0.480

*95% confidence limits

^#^no 95% confidence limits

### *In vitro* evaluation of ovicidal activity

**Fig 1:**
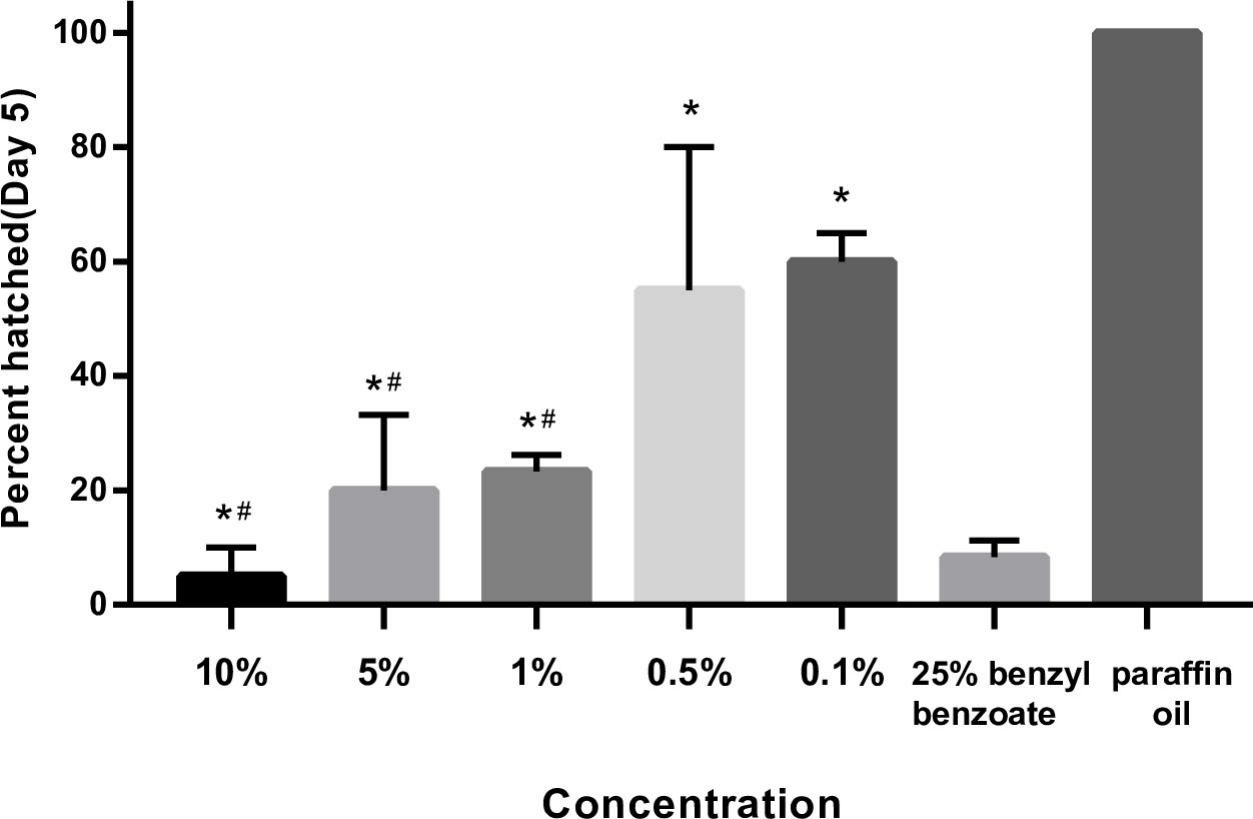
Hatching rates of *Sarcoptes* eggs exposed to lemongrass (*Cymbopogon citratus*) oil at the concentration of 10%, 5%, 1%, 0.5%, and 0.1% under laboratory conditions (35°C and ≥70% relative humidity). *Different concentrations of lemongrass oil compared to paraffin oil (negative control) (p<0.01). ^#^ Different concentrations of lemongrass oil compared to 25% benzyl benzoate (positive control) (p>0.05).

All *Sarcoptes* eggs in negative control groups hatched. The hatching rate of positive control groups was 8.3% (Fig 1). The hatching rate of *Sarcoptes* eggs exposed to lemongrass oil at the concentration of 10%, 5%, 1%, 0.5%, and 0.1% was 5%, 20%, 23.3%, 55%, and 60%, respectively. Lemongrass oil at all concentrations led to significant decrease in *Sarcoptes* egg hatching compared to the negative control (P<0.01). There is no significant difference between the positive control and the lemongrass oil at concentrations of 10% (p = 0.719), 5% (p = 0.220) and 1% (p = 0.121).

## Discussion

The chemical profile of lemongrass oil analysis in this study was comparable with that described previously from plants collected in Cameroon [19]. The results revealed a large proportion of citral, which is a natural mixture of two isomeric aldehydes, geranial (trans-citral, citral A) and neral (cis-citral, citral B) [20]. Previous reports showed that citral possesses antifungal activity against *Candida* yeasts [21] and anti-protozoan activity against *Leishmania amazonensis* [20]. A study demonstrated that citral has the best ovicidal and miticidal activity against *Tetranycchus urticae*, the two-spotted spider mite, compared to 33 commercial essential oils and compounds [16].

Paraffin oil is considered to be a satisfactory solvent for dissolving essential oils in bioassay. In the current study, all *Sarcoptes* mites were live after 24 h immersed in paraffin oil and all eggs hatched when only treated with paraffin oil. In a study of monoterpenes against ear mite (*Otodectes cynotis*), Olivier [22] tested three solvents (acetone, paraffin oil and 20% glycerol), demonstrating that paraffin oil not only exhibited good solubilization capability, good viscosity and volatility properties, but also induced the longest mean survival time of mites.

The present study revealed that lemongrass oil exhibited a significant concentration dependent miticidal effect against all life-stages of *S*. *scabiei*. Lemongrass oil at 10% and 5% concentration killed all mites within 10 min and 25 min, respectively. In a previous study, the lethal time of 10% and 5% tea tree oil was reported to be 30 and 90 min, respectively [10]. The results indicated that lemongrass oil could be comparable if not better than tea tree oil when used as miticide.

There was a significant decrease in hatching rate of *Sarcoptes* eggs exposed to all concentrations of lemongrass oil. The ovicidal activity of different essential oils has been demonstrated against the eggs of head lice (*Pediculus humanus capitis*) [23,24] and poultry red mite (*Dermanyssus gallinae*) [25]. A study evaluated the ovicidal activity of lemongrass oil against *Haemonchus contortus* (a nematode parasite of ruminants) eggs, resulting in a EC_50_ value of 0.14 mg/mL [26]. To the authors’ knowledge, the present study is the first to report the investigation of the ovicidal activity of an essential oil against *Sarcoptes* eggs. Since few available miticides possess ovicidal efficacy against *Sarcoptes* mites [5], lemongrass oil could be particularly beneficial in the control of scabies as it would reduce the need for multiple treatments to kill newly hatched larvae.

When used as a topical treatment, potential side effects of essential oils should also be considered. A review on patch tests demonstrated that 1.8% of the participants had positive allergic reactions to 2% of lemongrass oil [27]. Lemongrass oil is a mixture (Table 1), no study has clearly showed which compounds are responsible for the allergic reactions. Compounds such as geraniol and citronellol have been identified as potential skin sensitizers. However, both geraniol and citronellol were considered as less important or weak sensitizer in terms of frequency of sensitization [28,29] and both compounds accounted for very small percentage (1.55% and 1.10%) in the whole oil. According to the results of our study, lemongrass oil at the concentration of 5% is effective against both mites and eggs. A comprehensive review showed that the threshold for citral (a major component accounting for 69.37% in the present study) to induce dermal sensitization in human is 1.4 mg/cm^2^ [30], which guaranteed the safe usage of lemongrass oil (1 mL of 5% lemongrass oil contain about 35 μg citral) for this purpose.

A limitation is that all the tests in the present study were performed using *S*. *scabies* var. *cuniculi* instead of var. *hominis*. However, obtaining enough mites from patients with common scabies to perform *in vitro* tests is not feasible. Experimental models of rabbit and pig are suitable for the collection of large number of mites and they are well acceptable models for research purpose [10]. The single species *S*. *scabiei* infests different mammalian hosts in 17 families and 7 orders including humans and rabbits. Mites from different hosts may exhibit little differences regarding their morphology and host preference but they share the same biology [31]. From previous studies, it can be postulated that there was no difference in the survival rate of mites from rabbits and humans under the condition tested.

Currently, topical permethrin and oral ivermectin are the two most commonly used treatments for scabies [32]. However, both drugs are only available for scabies in a limited number of countries over the world, and both drugs are expensive [2,33]. Scabies is epidemic in tropical developing countries, where overcrowding and poverty are common, and where there is limited access to treatment [34]. In conjunction with education about its limitations and possible side effects, lemongrass oil, which extracted from a plant widely grown in tropical regions, should be considered a promising miticide against scabies, considering that it possesses significant activity against both *Sarcoptes* mites and eggs. Further studies should consider testing the efficacy of isolated main components against *Sarcoptes* mites and eggs to identify active compounds. The mechanism of action of active compounds is also worth investigating. Moreover, *in vivo* studies are necessary to confirm the efficacy and safety of lemongrass oil which could accelerate its use in scabies management.
